# Oxygenated Water Inhibits Adipogenesis and Attenuates Hepatic Steatosis in High-Fat Diet-Induced Obese Mice

**DOI:** 10.3390/ijms21155493

**Published:** 2020-07-31

**Authors:** Yuh-Jen Cheng, Chao-Chi Liu, Fang-Yeh Chu, Ching-Ping Yang, Chiao-Wan Hsiao, Cheng-Wei Chuang, Ming-Yuh Shiau, Hsueh-Te Lee, Jen-Ning Tsai, Yih-Hsin Chang

**Affiliations:** 1 Research Center for Applied Sciences, Academia Sinica, Taipei 115, Taiwan; yjcheng@sinica.edu.tw; 2 Department of Biotechnology and Laboratory Science in Medicine, National Yang-Ming University, Taipei 112, Taiwan; wiz0324@hotmail.com (C.-C.L.); d49407012@ym.edu.tw (C.-P.Y.); brianchuang0206@gmail.com (C.-W.C.); 3 Department of Clinical Pathology, Far Eastern Memorial Hospital, New Taipei City 220, Taiwan; jacpha@mail.femh.org.tw; 4 Graduate School of Biotechnology and Bioengineering, Yuan Ze University, Taoyuan 320, Taiwan; 5 Department of Medical Laboratory Science and Biotechnology, Yuanpei University, Hsinchu 300, Taiwan; 6 School of Medical Laboratory Science and Biotechnology, Taipei Medical University, Taipei 110, Taiwan; 7 Program in Molecular Medicine, National Yang-Ming University and Academia Sinica, Taipei 112, Taiwan; hcw_alicia_1990@hotmail.com; 8 Department of Nursing, College of Nursing, Hungkuang University, Taichung 433, Taiwan; ming@sunrise.hk.edu.tw; 9 Institute of Anatomy and Cell Biology, School of Medicine, National Yang-Ming University, Taipei 112, Taiwan; incubator.lee@ym.edu.tw; 10 Department of Medical Laboratory and Biotechnology, Chung Shan Medical University, Taichung 402, Taiwan; jeningts@csmu.edu.tw; 11 Clinical Laboratory, Chung Shan Medical University Hospital, Taichung 402, Taiwan

**Keywords:** oxygenated water, adipogenesis, adipocytes, hepatic steatosis, lipid metabolism

## Abstract

The expansion of adipose tissue mass is the primary characteristic of the process of becoming obesity, which causes chronic adipose inflammation and is closely associated with type 2 diabetes mellitus (T2DM). Adipocyte hypertrophy restricts oxygen availability, leading to microenvironmental hypoxia and adipose dysfunction. This study aimed at investigating the effects of oxygenated water (OW) on adipocyte differentiation (adipogenesis) and the metabolic function of mature adipocytes. The effects of OW on adipogenesis and the metabolic function of mature adipocytes were examined. Meanwhile, the in vivo metabolic effects of long-term OW consumption on diet-induced obesity (DIO) mice were investigated. OW inhibited adipogenesis and lipid accumulation through down-regulating critical adipogenic transcription factors and lipogenic enzymes. While body weight, blood and adipose parameters were not significantly improved by long-term OW consumption, transient circulatory triglyceride-lowering and glucose tolerance-improving effects were identified. Notably, hepatic lipid contents were significantly reduced, indicating that the DIO-induced hepatic steatosis was attenuated, despite no improvements in fibrosis and lipid contents in adipose tissue being observed in the OW-drinking DIO mice. The study provides evidence regarding OW’s effects on adipogenesis and mature adipocytes, and the corresponding molecular mechanisms. OW exhibits transient triglyceride-lowering and glucose tolerance-improving activity as well as hepatic steatosis-attenuating functions.

## 1. Introduction

Studies regarding oxygen concentration and diseases are emerging, such as the effects of oxygen contents and hypoxia on physiological functions and tissue damage. Abundant evidence shows that the rapid expansion of the major energy reservoir, adipose tissues, in obese individuals leads to a shortage of oxygen supply, which successively results in insulin resistance and type 2 diabetes mellitus (T2DM) [1]. Results from an in vitro study demonstrate that 3T3-F442A adipocytes produce large amounts of leptin and vascular endothelial growth factor A (VEGFA) under hypoxia [2], and 3T3-L1 adipocytes are induced to secrete numerous pro-inflammatory adipokines in a hypoxic environment [3]. As for in vivo studies, the epididymal adipose tissue of obese mice has lower oxygen pressure than that of mice with normal body weight [4]. In addition, white adipose tissues in leptin-deficient mice have a hypoxic status [1]. These reports reveal that obesity leads to adipose tissue hypoxia and contributes to adipose dysfunction.

As a matter of fact, a correlation between hypoxia and cytokine secretion was documented before the hypoxia of expanding adipose tissue had received significant attention. In response to the hypoxic stimulation, adipocytes undergo dramatic alterations of genetic expression and release pro-inflammatory adipokines such as tumor necrosis factor-alpha (TNF-α) and interleukin-6 (IL-6) [5]. These overproduced cytokines are the major mediators that promote inflammation and the development of insulin resistance [6].

Hypoxia-inducible factors (HIFs) are a family of proteins that function as important signaling molecules in response to hypoxia. HIF-1 plays the most critical role in regulating cellular responses under a hypoxic environment [7]. HIF-1 is a heterodimer composed of α and β subunits. While HIF-1β is constitutively expressed, the HIF-1α level is regulated by the environmental oxygen concentration [8]. HIF-1α is degraded after being associated with von Hippel-Lindau (VHL) under normoxia, whereas the binding between HIF-1α and VHL is down-regulated, which results in consistent HIF-1α expression, under hypoxic stimulation [9].

HIF-1α regulates the expression of multiple genes [10]. Hypoxia-induced HIF-1α accumulation in adipocytes is implicated in chronic inflammation, tissue fibrosis, adipose dysfunction and insulin resistance [11,12,13]. On the contrary, the disruption of adipose HIF-1α improves insulin sensitivity and decreases adiposity in diet-induced obesity (DIO) mice [14]. The abovementioned studies demonstrate that hypoxia-induced HIF-1α and the resultant inflammation participate in the development of insulin resistance and, therefore, are closely associated with metabolic disorders such as obesity and T2DM.

In the context that HIF-1α knockout reduces the tumorigenicity and metastasis of cancer cells [15], HIF-1α has been a target molecule for the development of cancer therapy [16]. Nevertheless, studies regarding HIF-1α, obesity and metabolic abnormalities are relatively limited. Accordingly, this study aimed at investigating the effects and possible regulation of adipocyte HIF-α by oxygenated water (OW) under a hypoxic microenvironment. Meanwhile, the possible improvement of hypoxia-induced negative influences on energy metabolism was also examined in vivo.

## 2. Results

### 2.1. Oxygenated Water Inhibits Adipogenesis

**Figure 1:**
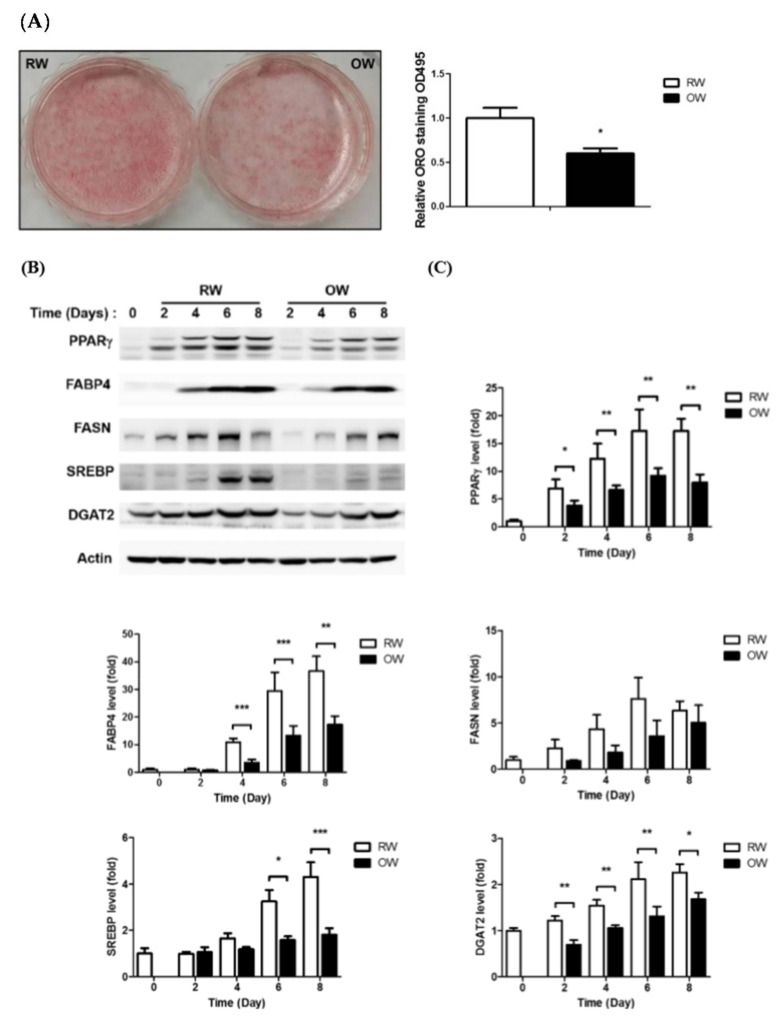
Oxygenated water inhibits adipocyte differentiation. 3T3-L1 cells were allowed to undergo differentiation in culture media with either regular water (RW) or oxygenated water (OW). (**A**) Lipid accumulation in 3T3-L1 mature adipocytes was evaluated and quantified by ORO staining. Data are expressed as mean ± S.E.M. (*n* = 3), * *p* < 0.05. (**B**) Levels of major proteins involved in adipocyte differentiation and lipid metabolism during differentiation were analyzed by Western blotting and are quantified in (**C**). Data are presented as mean ± S.E.M. (*n* = 4) and were statistically analyzed by two-tailed unpaired Student t-tests; * *p* < 0.05, ** *p* < 0.01, *** *p* < 0.001 (OW vs. RW).

Adipocyte differentiation is the process of pre-adipocytes becoming mature adipocytes through dramatic morphological changes and lipid accumulation [17]. Rapid and persistent lipid accumulation leads to obesity and related disorders [18]. Accordingly, manipulating and inhibiting adipocyte differentiation is a putative strategy for alleviating the consequences resulting from becoming obese. We hypothesized that an ambient oxygen supply would attenuate the hypoxia caused by rapid adipose expansion; therefore, the effects of OW treatment on the adipogenic efficiency of 3T3-L1 pre-adipocytes were examined. Interestingly, the lipid contents of cells cultured in OW were significantly decreased by about 40% on Day 8 (Figure 1A).

In addition, the protein levels of the central adipogenesis-driving transcription factor peroxisome proliferator-activated receptor-gamma (PPARγ) and adipocyte-specific marker fatty acid-binding protein 4 (FABP4) were significantly inhibited in cells under OW exposure. Critical lipid-synthesizing enzymes—including fatty acid synthase (FASN), sterol regulatory element-binding transcription factor 1 (SREBP) and diacylglycerol O-acyltransferase 2 (DGAT2)—were also down-regulated (Figure 1B,C). The above results indicate that OW significantly suppresses adipogenesis by down-regulating the expression of the critical transcription factor PPARγ, adipocyte-specific FABP4 and lipid-synthesizing enzymes required for lipid accumulation.

### 2.2. Effects of Oxygenated Water on Inflammatory Signaling in Mature Adipocytes

TNF-α is a cytokine with multiple functions such as regulating inflammation, apoptosis and energy homeostasis. Enhanced TNF-α production during the process of becoming obese is closely associated with metabolic abnormalities [19]. Inhibiting TNF-α antagonizes the adverse effects caused by obesity [20]. In this context, the effects of OW on TNF-α-induced inflammation in mature adipocytes were analyzed next.

**Figure 2:**
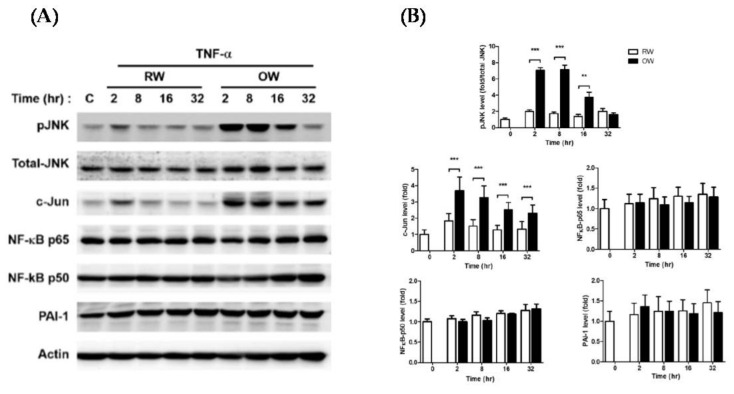
Effects of oxygenated water on inflammatory signaling and oxidative stress in mature adipocytes. 3T3-L1 mature adipocytes were pre-incubated in serum-free medium for 3 h, then treated with either OW or RW and 10 ng/mL of TNF-α for different time intervals. (**A**) Protein expression profiles of molecules involved in pro-inflammatory signaling were analyzed and quantified (**B**). Data are presented as mean ± S.E.M. (*n* = 4) and were statistically analyzed by two-tailed unpaired Student t-tests; ** *p* < 0.01, *** *p* < 0.001.

The TNF-α-mediated inflammatory JNK/c-Jun and NF-κB signaling pathways were analyzed in mature adipocytes with concurrent TNF-α and either OW or RW treatment for differential time intervals as indicated. The protein levels of JNK/c-Jun were rapidly increased by about 2 fold, while NF-κB and PAI-1 remained at consistent levels in TNF-α-treated cells under an RW environment (Figure 2). The TNF-α-elevated JNK/c-Jun expression was further increased in cells with concomitant OW exposure, reaching maximal levels at 2–8 h and gradually decreasing thereafter. However, no prominent alterations of NF-κB and PAI-1 levels were identified in the cells with combined TNF-α/OW treatment. The results suggest that TNF-α-induced adipose inflammation is mainly mediated via the JNK/c-Jun pathway rather than NF-κB signaling. Besides, OW promotes TNF-α-induced JNK/c-Jun inflammatory signaling in mature adipocytes.

### 2.3. Effects of Oxygenated Water on HIF-1α and Oxidative Stress

HIF-1α plays critical roles in the fibrosis of the expanding adipose tissue, thus contributing to adipose hypoxia and dysfunction [21]. We next investigated if OW would inhibit hypoxia-induced HIF-1α expression and ameliorate adipose dysfunction.

**Figure 3:**
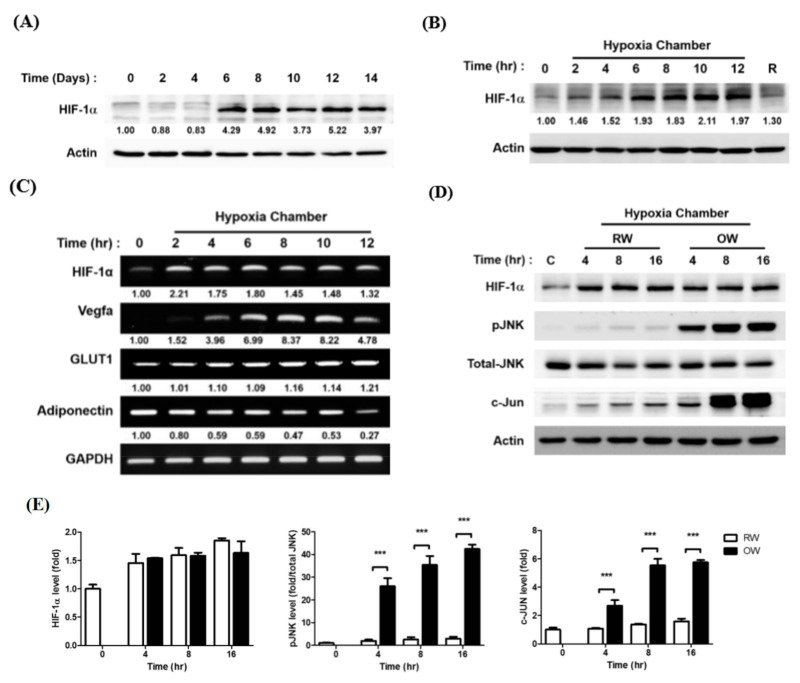
Effects of oxygenated water on hypoxia-induced HIF-1α expression. (**A**) Temporal expression pattern of HIF-1α protein during 3T3-L1 adipocyte differentiation, examined by Western blotting. Temporal expression pattern of HIF-1α proteins (**B**) and (**C**) mRNA after the differentiated mature adipocytes were pre-incubated in serum-free medium for 3 h and exposed to 1% O_2_ for different time intervals. R lane represents the cells being transferred back to normoxia (21% O_2_) for 16 h after 8 h of hypoxia treatment. mRNAs of HIF-1α downstream molecules were also examined (**C**). (**D**) Protein expression profiles of pro-inflammatory molecules after the mature adipocytes were pre-incubated in serum-free medium for 3 h, then exposed to 1% O_2_ with either RW or OW for different time intervals were analyzed (**E**). Data are presented as mean ± S.E.M. (*n* = 3) and were statistically analyzed by two-tailed unpaired Student t-tests, *** *p* < 0.001.

HIF-1α protein expression during the adipogenic process was first examined. HIF-1α was barely detected in pre-adipocytes (Day 0) but rapidly increased and peaked on Day 6–8 then was maintained at a high level in mature adipocytes until Day 14 (Figure 3A). Intriguingly, the timeline of high HIF-1α protein expression was coincident with the timeframe of lipid synthesis and accumulation in the differentiating cells. Next, the HIF-1α protein levels in mature adipocytes under a hypoxic environment were temporally examined. Likewise, HIF-1α rapidly increased and remained at a high level once the adipocytes were exposed to hypoxia (Figure 3B). The finding supports the idea that a hypoxic environment would be generated during the process of hyperplasia and hypertrophy in the rapidly expanding adipose tissues. The hypoxia-induced HIF-1α was attenuated by re-exposing the cells to a normoxic environment (R). The HIF-1α downstream VEGFA and glucose transporter 1 (GLUT1) mRNA amounts were also significantly increased during the period of hypoxia exposure (Figure 3C). On the contrary, the insulin sensitivity-promoting adiponectin was gradually decreased during hypoxia treatment, indicating the anti-inflammatory capacity of adipose tissue is reduced when it is expanding.

The effects of OW on hypoxia-induced HIF-1α were subsequently analyzed to evaluate the ability of OW to rescue the hypoxia-induced stress. While the hypoxia-induced HIF-1α protein level was not significantly altered, the activation of JNK/c-Jun signaling was dramatically enhanced by OW (Figure 3D,E). Accordingly, although OW suppresses adipocyte differentiation and lipid accumulation, it is insufficient for attenuating hypoxia-induced HIF-1α. On the contrary, OW further augments the JNK/c-Jun signaling pathway to enhance the inflammatory response.

**Figure 4:**
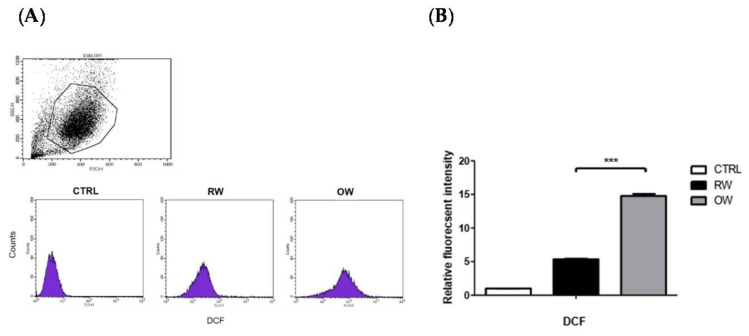
Effects of oxygenated water on oxidative stress in mature adipocytes. Mature adipocytes were pre-incubated with either OW or RW for 4 h then stained with the fluorescent dye (H_2_DCFDA) for 30 min. Fluorescent intensity of the H_2_DCFDA was detected by flow cytometry (**A**) and quantified (**B**). Data are presented as mean ± S.E.M. (*n* = 4) and were statistically analyzed by two-tailed unpaired Student t-tests, *** *p* < 0.001.

We speculated that the oxidative stress in the mature adipocytes may be increased by OW, thus triggering the activation of JNK/c-Jun signaling. Therefore, the intracellular ROS in OW-treated adipocytes under normoxia were analyzed. Intracellular ROS convert the non-fluorescent molecule 2′,7′-dichlorodihydrofluorescein diacetate (H_2_DCFDA) to the highly fluorescent 2′,7′-dichlorofluorescein (DCF), which can be detected by flow cytometry to indicate the original ROS amounts. The DCF intensity was examined after the mature adipocytes were incubated in either an OW or RW environment for 4 h. The DCF intensity was significantly elevated by about 3 fold in OW cells compared to in their RW counterparts (Figure 4). This indicates that OW enhances oxidative stress in mature adipocytes.

Taking the above in vitro findings together, while OW treatment inhibits adipogenesis, it is not sufficient for attenuating hypoxia-induced HIF-1α protein expression. Besides, OW increases oxidative stress to trigger the inflammatory JNK/c-Jun signaling under a normoxic environment and enhances the HIF-1α-mediated JNK/c-Jun pathway activation in response to hypoxic exposure.

### 2.4. Effects of Long-Term Oxygenated Water Consumption on Biochemical Parameters of Diet-Induced Obesity

**Figure 5:**
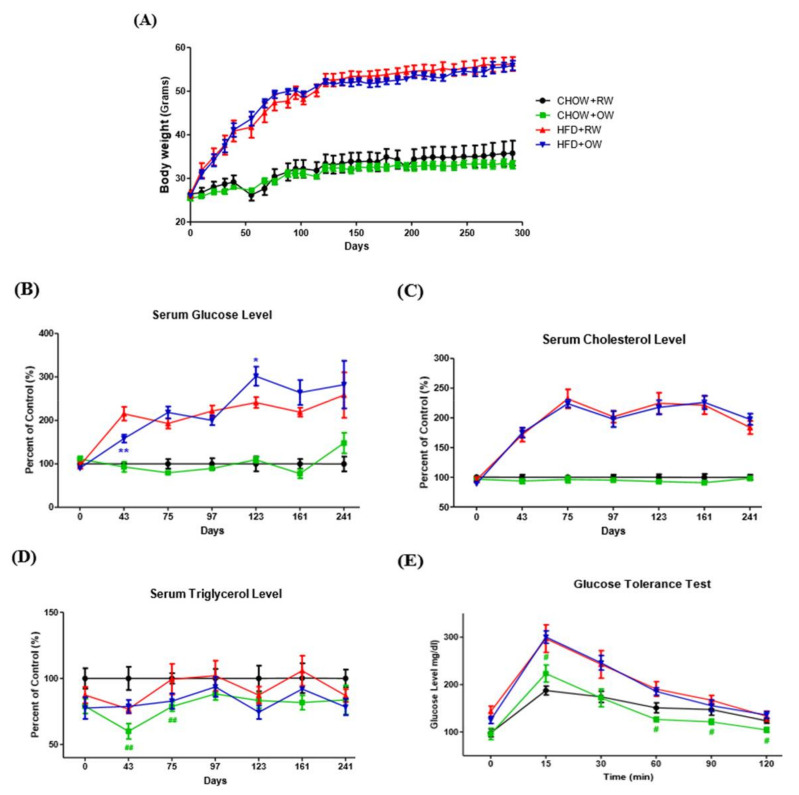
Effects of long-term oxygenated water consumption on biochemical parameters of diet-induced obesity. C57BL/6J mice were fed with a chow diet (CHOW) or high-fat diet (HFD) and provided with either OW or RW as drinking water. Total body weights (**A**) were continuously measured and documented during the entire study period. (**B**–**D**) Serum levels of glucose (**B**), total cholesterol (**C**) and triglycerol (**D**) were also continuously measured at the times indicated after 16 h of fasting. * *p* < 0.05, ** *p* < 0.01 (HFD+OW vs. HFD+RW), ^##^ *p* < 0.01 (CHOW+OW vs. CHOW+RW). (**E**) Pattern of plasma glucose during glucose tolerance test (GTT) after 16 h of fasting, ^#^ *p* < 0.05 (CHOW+OW vs. CHOW+RW). Data are presented as mean ± S.E.M. (*n* = 5) and were statistically analyzed by two-tailed unpaired Student *t*-tests.

We next examined the in vivo effects of long-term OW consumption on energy metabolism. Body weights were continuously monitored in C57BL/6 mice on either a high-fat diet (HFD) or regular chow diet (CHOW), providing OW or RW as drinking water, for about 300 days. The HFD mice had significantly higher body weights, which indicated that the diet-induced obesity (DIO) model was successfully established by the HFD. However, no prominent changes in body weights between the OW-drinking mice and their RW counterparts were observed during the entire study period (Figure 5A).

Blood biochemical parameters were analyzed during the entire study period. While long-term OW consumption did not improve fasting blood glucose and cholesterol profiles, a short-term fasting glucose- and TG-lowering effect was identified in HFD+OW (43 days) and CHOW+OW mice (43–75 days), respectively (Figure 5B–D). Notably, the time required for the elevated glucose to return to euglycemic levels in the glucose tolerance test in CHOW+OW mice was significantly shorter than that in their RW counterparts (Figure 5E). These findings demonstrate that OW shows short-term TG-lowering activity and tends to improve glucose tolerance under physiological conditions.

### 2.5. Effects of Long-Term Oxygenated Water Consumption on Adipose Tissue

**Figure 6:**
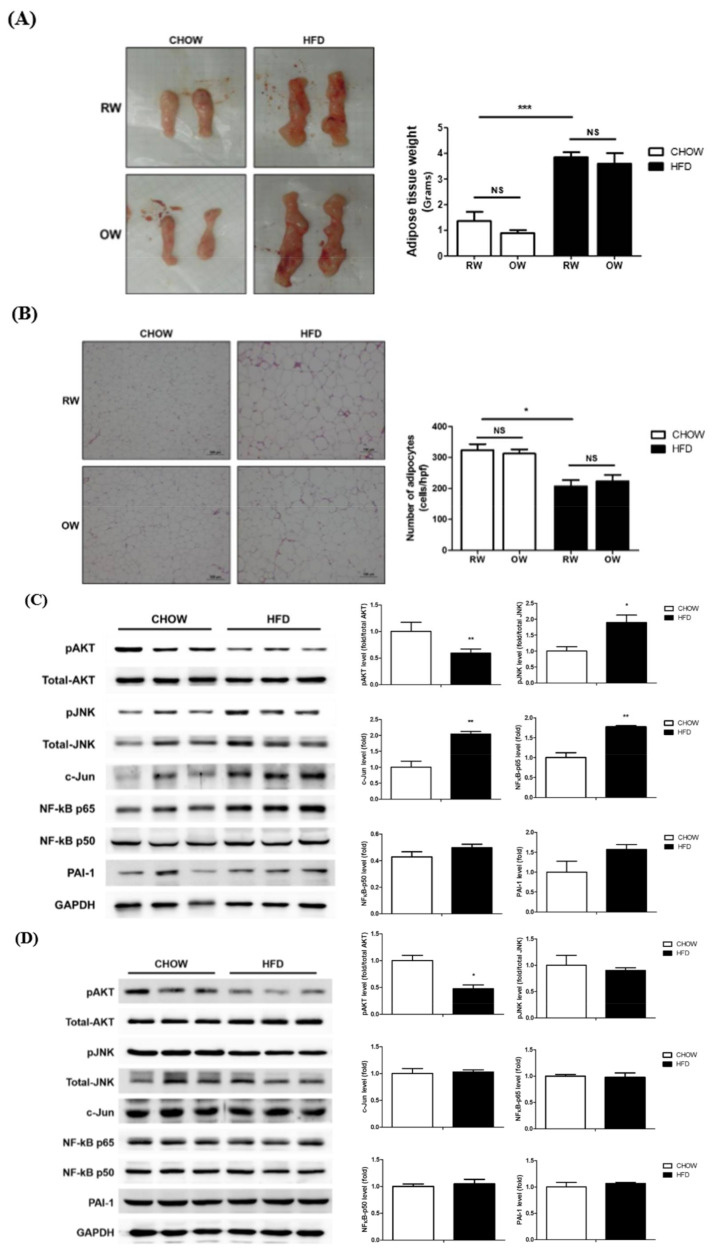

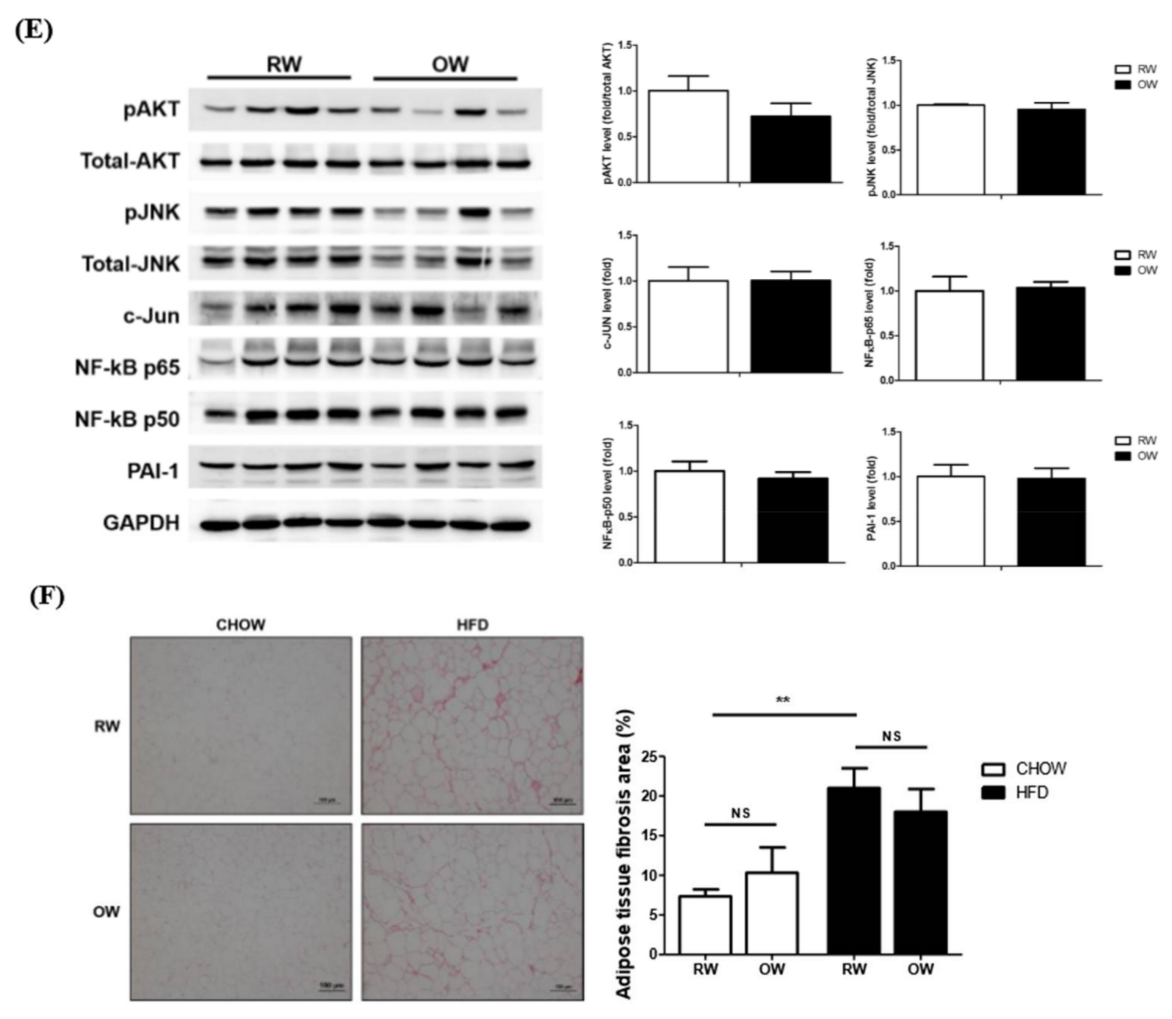
Effects of long-term oxygenated water consumption on muscle and adipose tissues. C57BL/6J mice were fed with CHOW or HFD and either OW or RW as drinking water. (**A**) Weights of epididymal white adipose tissue (eWAT) were measured at the end of study period. (**B**) Representative results of eWAT H&E staining. Number of adipocytes were calculated and presented as number of cells/high power field (400×), scale bar: 100 μm. (**C**,**D**) Levels of pro-inflammatory molecules protein and pAKT in eWAT (**C**) and muscle (**D**) of CHOW and HFD mice were analyzed and quantified. (**E**) Levels of pro-inflammatory molecules protein and pAKT in eWAT were analyzed. (**F**) Collagen accumulation in eWAT were determined by sirius red (SR) stain, scale bar: 100 μm. Degree of fibrosis was semi-quantified by MetaMorph software. Data were presented as mean ± S.E.M (*n* = 5), and statistically analyzed by two-tail unpaired Student *t*-test, * *p* < 0.05, ** *p* < 0.005, *** *p* < 0.001, NS: no significance.

As the inflammation of rapidly expanding adipose tissue during the process of becoming obese is an important factor leading to insulin resistance and metabolic abnormalities, the putative alleviating effects of OW on the DIO consequences in HFD mice were investigated. No significant alterations regarding the wet weights (Figure 6A) and adipocyte cross-sectional area (Figure 6B) of epididymal white adipose tissue (eWAT) were identified between the HFD and HFD+OW mice. The HFD significantly reduced the levels of the master insulin-signaling molecule pAkt while enhancing JNK/c-Jun and NF-κB p65 activation in adipose tissue, compared with that in their CHOW counterparts (Figure 6C). Intriguingly, the HFD also affected pAkt levels in muscle, while the pro-inflammatory JNK/c-Jun and NF-κB p65 signaling molecules remained unchanged (Figure 6D). OW did not attenuate the HFD-induced changes in the amounts of these signaling molecules (Figure 6E). These findings indicate that DIO impairs adipose tissue insulin signaling and induces inflammation. However, long-term OW consumption is not sufficient for ameliorating these affected adipose behaviors. Besides, no inflammatory pathways are activated in myocytes, while DIO also disturbs muscle insulin signaling.

In addition to chronic inflammation, fibrosis is considered to be an important cause of adipose functional dysregulation [22]. Therefore, collagen accumulation in eWAT was analyzed to evaluate the degree of adipose fibrosis. The HFD significantly induced prominent adipose fibrosis, while OW did not rescue the HFD-induced fibrosis (Figure 6F). Taking the above data together, it is apparent that OW is not sufficient for alleviating the DIO-induced impairment of adipose insulin signaling, fibrosis and chronic inflammation.

### 2.6. Long-Term Oxygenated Water Consumption Ameliorates Hepatic Steatosis

**Figure 7:**
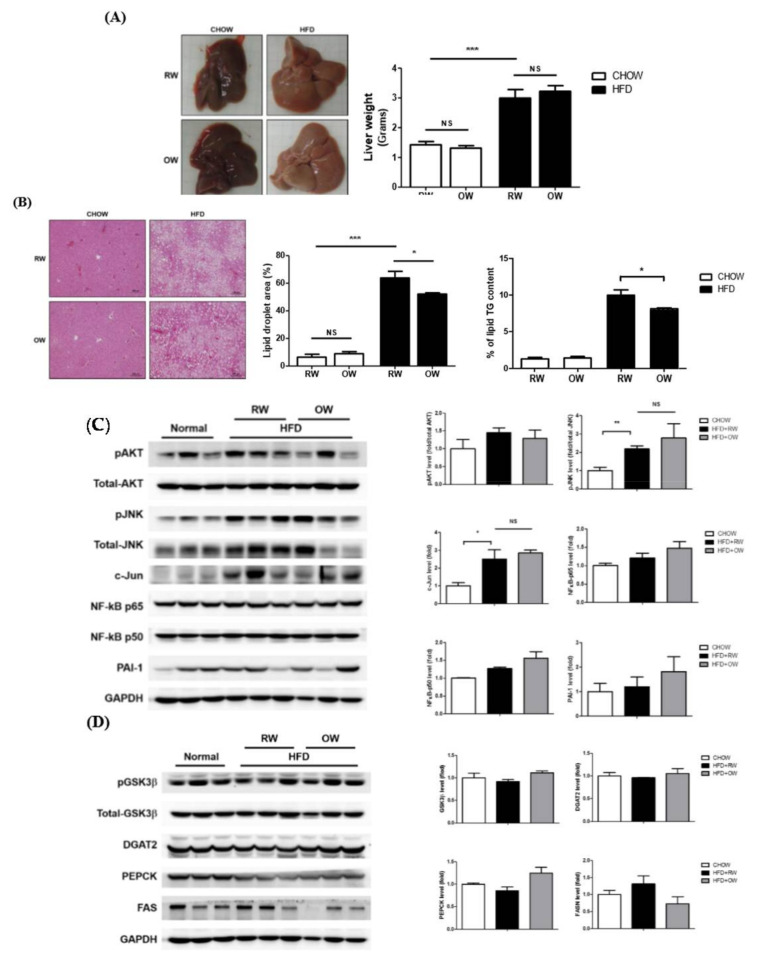
Long-term oxygenated water consumption ameliorates hepatic steatosis. C57BL/6J mice were fed with CHOW or HFD and either OW or RW as drinking water. (**A**) Liver weights were measured at the end of study. (**B**) Hepatic contents of lipid and triglyceride (TG) were determined as detailed in Methods, scale bar: 100 μm. Levels of hepatic pro-inflammatory molecules and pAKT (**C**) as well as major proteins in involved in energy metabolism (**D**) were analyzed and quantified. Data were presented as mean ± S.E.M, and statistically analyzed by two-tail unpaired Student *t*-test; * *p* < 0.05, ** *p* < 0.01, *** *p* < 0.001, NS: no significance.

Fatty liver is closely associated with obesity, with a prevalence about 76% in the obese population and 50% in diabetes patients. Therefore, we intended to examine if drinking OW would alleviate fatty liver in DIO mice. Macroscopically, HFD led to hepatic steatosis and increased liver wet weights. No significant differences between OW-drinking mice and their corresponding counterparts were identified (Figure 7A). The area of lipid droplets and triglyceride (TG) contents were significantly increased in HFD mice drinking RW (Figure 7B). Notably, while no significant differences were observed in the liver weights between HFD and HFD+OW mice, hepatic lipid and TG contents were significantly reduced in OW-drinking HFD mice.

Pro-inflammatory mediators in liver tissue were next examined. As in the scenario in adipose tissue, JNK/c-Jun signaling was significantly induced in HFD mice, while NF-κB p65 and PAI-1 protein levels remained consistent (Figure 7C). The HFD-induced FASN protein expression was partially reversed, however, without a significant difference (Figure 7D). The protein amounts of critical hepatic enzymes participating in energy metabolism, including the glycogen synthesis-mediating glycogen synthase kinase-3 beta (GSK-3β), long chain fatty acid-synthesizing diacylglycerol O-acyltransferase 2 (DGAT2) and gluconeogenesis-regulating phosphoenolpyruvate carboxykinase (PEPCK), were not prominently altered by OW (Figure 7D). The above results reveal that, although OW does not improve the inflammatory responses in the liver, it shows lipid-lowering effects possibly through the down-regulation of FASN expression.

## 3. Discussion

Abdominal obesity is a major risk factor for metabolic abnormalities [18]. Adipose tissue is no longer considered as a passive organ, a mere energy-preserving reservoir for a rainy day. Instead, adipose tissue actively participates in regulating multiple physiological functions. The process of adipose tissue enlargement due to nutrition oversupply leads to consequences such as chronic inflammation, hypoxia, aberrant adipokine secretion and dysfunction [23,24]. Rapid adipose expansion has been implicated in the pathogenesis of T2DM and the related complications.

Adipogenesis is finely coordinated by sequential events, including the initial activation of transcription factors in the mitotic cell expansion (MCE) phase to increase cell numbers, followed by dramatic morphological alterations and lipid synthesis/accumulation for the cells to acquire the characteristics of mature adipocytes. Our in vitro data demonstrate that OW suppresses adipocyte differentiation by multiple regulatory functions.

First of all, OW down-regulates the expression of the adipogenesis-promoting transcription factor PPARγ on Day 2 in the early adipogenic phase, suggesting OW inhibits adipogenesis by targeting the MCE phase. Secondly, the critical lipid-synthesizing enzymes including FABP4, SREBP1 and DGAT2 were significantly decreased in OW-treated cells. This demonstrates that OW also exhibits inhibitory activity against lipid synthesis and accumulation, which leads to the significantly lower lipid contents in the differentiated mature adipocytes.

During the process of gaining body weight and becoming obese, adipose tissue faces multiple stresses such as inflammation and oxidative and ER stresses. These intracellular stresses elicit the activation of stress-sensing kinases and downstream signaling pathways, which are implicated in adipose dysfunction and metabolic imbalance and result in the onset of metabolic disorders [25]. In particular, the accumulated intracellular super-active ROS cause the functional impairment of proteins, carbohydrates and lipids, resulting in the release of even more ROS. This vicious cycle eventually leads to damage, the impairment of tissue function and various diseases.

TNF-α is one of the most extensively studied pro-inflammatory cytokines, which shows a prominent link to the onset of metabolic disorders [26] via inducing the obesity-associated HIF-1α and low-grade chronic inflammation of the adipose tissue [27,28,29]. Although OW significantly reduces adipocyte differentiation and lipid contents, the treatment is not sufficient for attenuating the TNF-α induced inflammatory signaling. On the contrary, our results demonstrate that OW increases the intracellular oxidative stress and further enhances TNF-α−elicited JNK/c-Jun signaling. On the other hand, OW does not attenuate hypoxia-induced HIF-1α expression and the elicited JNK/c-Jun signaling.

The application of hydrogen treatment in metabolic disorders is well-studied in animal and clinical settings. Many reports have confirmed that H_2_ consumption reduces oxidative stress in various disease models [30,31,32,33,34,35,36,37,38,39]. Absorbing hydrogen by natural respiration, injecting hydrogen-infused saline or drinking hydrogenated water (H_2_-water) shows efficacy in treating diabetes mellitus, atherosclerosis and hepatitis C [31]. Kamimura et al. documents that the liver accumulates the absorbed H_2_, and the hepatic oxidative stress and steatosis resulting from obesity are effectively inhibited in mice by the consumption of H_2_-water [40]. In addition, long-term H_2_-water consumption significantly controls fat and body weights. Not only are circulatory glucose and triglycerides significantly decreased but adipose hyperplasia is attenuated in DIO mice by the consumption of H_2_-water. Moreover, drinking H_2_-water promotes liver lipid and glucose metabolism by activating the hepatic expression of fibroblast growth factor 21. These findings suggest H_2_ has great potential to be developed as a therapeutic and/or preventive means for metabolic syndrome.

Unlike the scenario of H_2_-water, solid scientific evidence and the corresponding metabolic effects of OW on improving metabolism still await to be examined. The earliest literature documenting the study of OW dates back to the 1990s; evidence regarding OW’s effects has accumulated thereafter. The results showed that consuming OW did not significantly promote athletic performance [41]. Exercise strength, heart beats, the respiratory exchange rate and circulatory lactate levels were not significantly affected by drinking OW [42]. As for possible oxygen toxicity, OW does not cause significant damage to the hepatic, blood and immune systems. Despite ROS being transiently increased after drinking OW, the increase in ROS has been proved to be a time-limited and moderate generation of radicals. The regular and long-term consumption of OW seems to attenuate this effect [43]. Nevertheless, no molecular data were demonstrated to explain the underlying mechanism.

Hyperbaric oxygen therapy (HBOT) is a widely-used and effective treatment to improve tissue hypoxia and promote wound healing by rapidly elevating blood and tissue oxygen concentrations. Several studies demonstrate that HBOT harbors neuroprotective action by promoting neurogenesis and recovering impaired function in the treatment of acute stroke [44,45,46,47,48,49]. The HBOT-induced ROS act as a double-edged sword, enhancing the bactericidal ability of neutrophils but also leading to the secretion of multiple cytokines, elevated HIF-1α and oxidative stress [50,51,52,53]. The HBOT-enhanced ROS production may exert cytotoxic effects and is implicated in several complications such as neurodegenerative diseases, brain cell death, pulmonary damage and epilepsy [49]. Our data regarding the OW induction of ROS and JNK/c-Jun signaling in adipose tissue may explain the underlying molecular mechanisms of the elevated ROS in HBOT. Although accumulating evidence may aid HBOT development and application, the dose, duration and practical protocol of HBOT needed to achieve optimal therapeutic effects still await further study. Therefore, the efficacy and the underlying mechanism of O_2_ treatment need to be closely monitored and elucidated for HBOT practice in clinical settings.

The limitation of this study is that it did not provide quantitative data regarding how much OW or RW the mice drink per day, since the mice could freely approach the provided water. Nevertheless, this study provides evidence concerning the in vivo effects on metabolic homeostasis of long-term OW consumption. Besides, the effects of OW on liver function were not determined. However, Gruber et al. reveals that long-term OW consumption seems to be safe and has no adverse effects on hepatic function [41]. Except for inducing a moderate transient systemic increase in oxygen radicals, 28-day OW consumption shows no pathological effects on the liver, the blood or the immune system. In addition, the OW culture medium in the in vitro study needed to be frequently replaced by freshly-prepared medium with OW to keep the cells continuously exposed to a high O_2_ environment, since the dissolved O_2_ contents of the OW were decreasing with time due to diffusion. Therefore, the dose effects of OW with fixed O_2_ contents on adipogenesis were not examined.

In summary, the in vitro results in the present study demonstrate that OW suppresses adipocyte differentiation and lipid accumulation. The in vivo data reveal that while body weights and metabolic parameters are not improved by long-term OW consumption, transient circulatory triglyceride-lowering and glucose tolerance-improving effects are identified. Intriguingly, OW significantly reduces hepatic lipid contents and improves hepatic steatosis. It would be tempting to investigate whether long-term OW consumption would promote glucose tolerance and thus improve the efficacy of energy metabolism in human beings.

## 4. Materials and Methods

### 4.1. Reagents

Primary antibodies against β-Actin, AKT, phosphyorylated AKT (pAKT), GAPDH, glycogen synthase kinase-3 beta (GSK-3β), phosphorylated GSK-3β (pGSK-3β) Ser-9, HIF-1α, NF-κB p65, NF-κB p50, PAI-1 and phosphoenolpyruvate carboxykinase (PEPCK) were purchased from Abcam (Cambridge, UK); those against fatty acid synthase (FASN), fatty acid binding protein-4 (FABP4), pJNK and peroxisome proliferator-activated receptor gamma (PPARγ) were purchased from Cell Signaling (Danvers, MA, USA); those against JNK, c-Jun, sterol regulatory element-binding protein (SERBP1) and VEGFA were purchased from Santa Crutz; and that against diglyceride acyltransferase 2 (DGAT2) was purchased from Genetex. Secondary anti-rabbit antibody was purchased from Santa Cruz; anti-mouse antibody, from GeneTex; and anti-goat antibody, from Millipore (Temecula, CA, USA). Clarity^TM^ Western ECL Substrate was purchased from Bio-Rad; TRIzol Reagent, from Invitrogen; and 3-isobutyl-methylxanthine (IBMX), dexamethasone (Dex), and insulin, from Sigma.

### 4.2. Preparation of Saturated Oxygenated Water

The oxygenated water was prepared using a home-built apparatus. Oxygen gas and water were simultaneously supplied to the inlet of a diaphragm pump (HF-9200 HEADON) to compress oxygen into water under 5 kg/cm^2^ of pressure. The oxygen and water flow rates were 10 and 250 mL/min, respectively. The oxygen was fully dissolved in the water at the outlet of the diaphragm pump. The pressurized water was then released to atmospheric pressure by flowing it through a 150 μm diameter nozzle connected to the outlet. The dissolved oxygen in the water resulted in oversaturation due to pressure release. The excess oxygen formed bubbles, which were subsequently released into the air, leaving saturated oxygenated water (OW) at atmospheric pressure. The oxygen content of the OW was measured with a dissolved oxygen meter (YaLab DO-5512SD, Taipei, Taiwan). It was 100% saturation (40 mg/L) for the freshly prepared OW. The dissolved oxygen content decreased with time due to the diffusion of the dissolved oxygen into ambient air. It gradually decreased to 50% saturation (20 mg/L) in 12 h. The oxygen content further decreased to 30% (12 mg/L) saturation after another 12 h, and finally to 20% saturation (8 mg/L) in equilibrium with oxygen content in the air by the 40th hour after the initial OW preparation.

### 4.3. T3-L1 Cell Culture, Adipogenesis and Oil-Red O Staining

3T3-L1 pre-adipocytes were maintained in DMEM containing 10% calf serum (Hyclone Laboratories). Two-day post-confluent 3T3-L1 cells (designated Day 0) were induced to differentiate by the addition of a standard cocktail (MDI) composed of 0.5 mM IBMX, 1 μM Dex, and 10 μg/mL of insulin in 10% fetal bovine serum for 2 days. The cells were then cultured in DMEM supplemented with 10% FBS and 5 μg/mL of insulin for the next 6 days to allow the cells to become mature adipocytes. The medium was replaced by fresh medium every two days. For investigating the effect of OW on adipogenesis, culture media were prepared using freshly-prepared OW. Oil-Red O staining was performed as previously described [54,55,56]. For quantification, the dye was eluted by adding 100% isopropanol, and the extracts were analyzed by measuring the absorbance at 490 nm. For culturing the cells under a hypoxic environment, mature adipocytes were first cultured in serum-free media for 16 h then switched to the culture media prepared with either OW or regular water (RW) in a hypoxic chamber containing 1% O_2_/94% N_2_/5% CO_2_ at 37 °C. The cells were harvested at the indicated time.

### 4.4. Western Blot Analysis

Cell lysates were prepared as described previously [55,56,57,58]. In brief, protein extracts from epididymal fat tissues were obtained after the tissues were homogenized using T-PER tissue protein extraction reagent (Pierce, Rockford, IL, USA) supplied with phosphatase and protease inhibitors (Roche, Indianapolis, IN, USA). The protein lysates were normalized using Bio-Rad protein reagent and resolved by SDS-PAGE then electrotransferred to a PVDF membrane and blotted with specific primary antibodies. For detection, the membranes were incubated with secondary antibodies (Merck Millipore, Billerica, MA, USA) for 1 h, and the results were visualized with ECL regent and exposure to X-films. The blot was quantified using the Labscan software.

### 4.5. RNA Extraction and RT-PCR

Total RNA was isolated using TRIzol Reagent. Briefly, cDNA was synthesized using 5 μg of RNA, 200 pmol of oligo dT primer and 5× MMLV RT. Three microliters of first-strand cDNA was amplified using target sequence-specific PCR primer sets (HIF-1α: 5’-TTGCCACTTCCCCACAATGT-3’ and 5’-TGATGGTGAGCCTCATAACAGAA-3’; VEGFA: 5’-CGGGATTGCACGGAAACT-3’ and 5’-GGCTACTACGGAGCGAGAAGAG-3’; GLUT1: 5’-CGCCCCCCAGAAGGTTAT-3’ and 5’-TCCGTAGCGGTGGTTCCAT-3’; adiponectin: 5’-GCAAGCTCTCCTGTTCCTCTTAAT-3’ and 5’- GCTCTTCAGTTGTAGTAACGTCATCTTC-3’; and GAPDH: 5’- AGATGACCCAGATCATGTTTGAGA-3’ and 5’-CACAGCCTGGATGGCTACGT-3’; primer sequences designed by using the ABI primer expression software 3.0) with the GoTag Hot Start Green Master Mix (Promega). All RT-PCR reactions were carried out with the Bio-Rad PCR iCYCLER instrument. The amplified products were identified by electrophoresis on a 2% agarose gel. The quantitative results were presented as the means of three independent experiments.

### 4.6. Detection of Intracellular Reactive Oxygen Species (ROS)

ROS were measured with 2′,7′-dichlorodihydrofluorescein diacetate (H_2_DCF-DA; purchased from Life Technologies). Cells were first serum-starved from 4 h, switched to RW- or OW-containing media for another 4 h, then incubated with 200 μM H_2_DCF-DA for 30 min after PBS washing. The intracellular fluorescent intensity was analyzed by flow cytometry (BD FACSCalibur). Data are presented as the mean values from 10,000 cells and were analyzed with the FlowJo 7.6.1 software (FlowJo LLC, Ashland, OR, USA).

### 4.7. Animal Experiments

Four-week-old male C57BL/6 mice were randomly divided into 4 groups according to the feeding diet (with 13% fat-containing chow diet or 60% fat-containing high fat diet (HFD)) and drinking water (RW or OW). The drinking water in the OW groups was frequently replaced by freshly prepared OW. The animal protocols were reviewed and approved by the Institutional Animal Care and Use Committee, National Yang-Ming University (IACUC No. 1071103, 5 November 2018). The body weights and fasting biochemical parameters including glucose (GLU), total cholesterol (TCHO) and triacylglycerol (TG) were continuously analyzed with the Fujifilm (Kanagawa, Japan) DRI-CHEM4000i and recorded over the entire study period. The adipose, liver and muscle tissues were harvested for subsequent analysis.

### 4.8. Intraperitoneal Glucose Tolerance Test (IGTT)

The IGTT was performed, and blood glucose was measured using the OneTouch monitoring system (LifeScan, Milpitas, CA, USA) before and after *i.p* glucose injection (2 g/kg; Sigma-Aldrich, Steinheim, Germany) at the indicated time. The epididymal fat pads were taken and weighed, and the adipocytic cross-sectional areas from the staining images were calculated.

### 4.9. Histological Analysis

For hematoxylin and eosin staining, tissues were fixed in 10% formalin for 48 h. The tissues were dehydrated using ethanol and were cleared with xylene, after which they were embedded in paraffin and cut into 4–7 μm sections. Slides of the tissue sections were then deparaffinized, dehydrated and stained with hematoxylin and eosin. Collagen fibers were stained with picric acid sirius red (SR stain) and observed by high-resolution imaging using a slide fluorescence microscope equipped with cameras (Leica DM4000-B, Wetzlar, Germany).

### 4.10. Hepatic Lipid Contents and Triglyceride Assay

Hepatic lipid contents were determined by H&E staining and semi-quantified by measuring the areas of lipid droplets using the MetaMorph software. The triglyceride assay was conducted as described previously [56,59] with modifications. In brief, liver tissues were homogenized and heated to dissolve cellular triglycerides. Supernatants were collected after the homogenates were centrifuged at 13,000 *g* for 2 min, followed by incubating the samples and Triglyceride Assay Buffer in a 96-well microplate with 2 μL of lipase for 30 min then with Reaction Mix for another 30 min at room temperature. The triglyceride contents were determined according to the absorbance at 570 nm.

### 4.11. Statistical Analysis

Each experiment was carried out at least three times. The results are presented as mean ± S.E.M. and significant differences between groups were determined by two-tailed unpaired Student t-tests, or one-way or two-way ANOVA. Statistical significance was defined as *p* < 0.05 for all tests.

## 5. Conclusions

By integrating our data and findings from mice fed with a HFD for differential periods [54,55,56,57,58,59,60,61,62], it was demonstrated that adipose tissue and the liver are more susceptible to a HFD at the early phase in the sequential events of DIO leading to metabolic dysregulation. First of all, excess glucose and nutrients are stored as triglycerides in the liver and adipose tissue, causing organ-specific insulin resistance, hepatic steatosis and adipose enlargement at this stage. The abnormalities of lipid metabolism are manifested as elevated blood lipid panels (including cholesterol, triglycerides and free fatty acids), while fasting glucose and insulin levels remain within the normal reference ranges, despite glucose tolerance, insulin sensitivity and critical insulin signaling molecules (pAkt/pGSK-3β) being reduced. In the meantime, the distorted adipokine-producing pattern from hypertrophic adipose will release several inflammatory factors and adipokines into the circulation, actively affecting the metabolic efficiency of distal organs and tissues. In addition to there being steatosis, the increased serum ALT indicates that HFD triggers liver damage and chronic inflammation.

On the other hand, as one of the three major insulin-targeting tissues and the main glucose consumer, muscle metabolizes and transforms most of the taken up glucose to meet its energy demands. Besides, the present study reveals that no inflammatory pathways are activated in myocytes by long-term HFD, while the insulin signaling pathway is disturbed. Thus, the liver and adipose tissue not only are more susceptible to overnutrition but should contribute more to the deterioration of metabolic balance.

The progression of metabolic abnormalities would be possible to reverse if an appropriate intervention was adopted, e.g., OW administration in the present study or IL-4 to restore energy metabolic efficiency and attenuate adipose/hepatic adiposity before systemic insulin resistance is developed. On the contrary, once left untreated, the metabolic dysregulation of hepatic steatosis and hypertrophic adipose tissue under long-term overnutrition are implicated in the development of systemic insulin resistance and eventually metabolic disorders. In this context, information with regard to the manipulation of expanded adipose tissue and amelioration of hepatic steatosis sheds light on the development of anti-obesity strategies for tackling the increasing global prevalence of metabolic consequences and disorders.

## Abbreviations

Dex	dexamethasone
DGAT2	diacylglycerol O-acyltransferase 2
FABP4	Fatty Acid-Binding Protein 4
FASN	fatty acid synthase
GLU	glucose
GLUT	glucose transporter
HFD	high fat diet
HIFs	hypoxia-inducible factors
IBMX	3-isobuty-methylxanthine
IL-	interleukin-
OW	oxygenated water
PEPCK	phosphoenolpyruvate carboxykinase
PPARγ	peroxisome proliferator-activated receptor-gamma
p-	phosphorylated
RW	regular water
SREBP	sterol regulatory element-binding transcription factor 1
siRNA	small interfering RNA
T2DM	type 2 diabetes mellitus
TCHO	total cholesterol
TG	triacylglycerol
TNF-α	tumor necrosis factor-alpha
VEGFA	vascular endothelial growth factor A
VHL	von Hippel-Lindau
